# Attitudes towards vaccination in multiple sclerosis: insights from patients, neurologists, and general practitioners within a qualitative study

**DOI:** 10.1186/s12883-026-05590-1

**Published:** 2026-11-03

**Authors:** Anastasia Suslow, Romy Lauer, Thomas Grüter, Kerstin Hellwig, Chantal Flemm, Nina Timmesfeld, Jale Basten, Robin Denz, Marianne Tokic, Katharina Meiszl, Theresa Oganowski, Uta Kiltz, Maria Zacharopoulou, Andreas Stallmach, Anika Franz, Ursula Marschall, Joachim Saam, Catharina Tiemann, Kathrin J. Grochau, Horst Christian Vollmar, Ina Carola Otte

**Affiliations:** 1 Medical Faculty, Institute of General Practice and Family Medicine (AM RUB), Ruhr University Bochum, Bochum, 44801 Germany; 2 Department of Medical Informatics, Biometry and Epidemiology, Ruhr University Bochum, Bochum, Germany; 3 Department of Neurology, St. Josef Hospital, Ruhr University Bochum, Bochum, Germany; 4 Department of Neurology and Stroke Unit, Klinikum Lippstadt, Lippstadt, Germany; 5 Department of Geriatric Medicine, Marien Hospital Herne, Ruhr University Bochum, Herne, Germany; 6 Rheumazentrum Ruhrgebiet, Ruhr University Bochum, Herne, Germany; 7 Department of Internal Medicine IV (Gastroenterology, Hepatology and Infectious Diseases), Jena University Hospital, Jena, Germany; 8 Department Medicine and Health Services Research, BARMER Institute for Health System Research, Wuppertal, Germany; 9 Department of Health Services Research, Institute of Diversity Medicine, Ruhr University Bochum, Bochum, Germany

**Keywords:** Immunization, Vaccination hesitancy, Multiple sclerosis (MS), Doctor-patient communication, Infection risk

## Abstract

**Background:**

People with Multiple Sclerosis (pwMS) face increased infection and mortality risks due to their disease and potentially immunosuppressive therapy. Comprehensive vaccination is recommended, but reported vaccination rates in pwMS vary, and concerns about safety und effectiveness are discussed as one possible reason.

**Objectives:**

This study examines the vaccination rates and hesitancy in pwMS from the perspectives of patients, neurologists, and general practitioners (GPs) to identify possible approaches to overcome the hesitation.

**Methods:**

Between March and December 2023 narrative, semi-structured interviews were conducted with fifteen patients, five neurologists, and ten GPs in Germany. Pseudonymized data were analyzed using Kuckartz’s Qualitative Content Analysis with MAXQDA, incorporating deductive and inductive codes.

**Results:**

In total, thirty participants were interviewed. Our findings suggest that some pwMS experience gaps in vaccination-related information and desire more proactive information from physicians. Several participants described that communication between neurologists and GPs was limited and that it was not always clear which physician addresses vaccination counselling and administration. Fear of side effects and misinformation encountered by patients affect vaccination willingness. Patients prefer precise and consistent information materials on vaccination timelines.

**Conclusion:**

Misinformation or lack of information may contribute to vaccination reluctance. Addressing patients’ concerns with comprehensible information appears to be an important element of vaccination counselling from the perspective of the participants. Our findings highlight the importance of improved communication and education among all parties involved in vaccination.

**Trial registration:**

German Register of Clinical Studies (DRKS): https://drks.de/search/de/trial/DRKS00031559 (Registration Date: 28.08.2023, retrospectively registered).

**Supplementary Information:**

The online version contains supplementary material available at https://doi.org/10.1186/s12883-026-05590-1.

## Introduction

Patients with autoimmune diseases may face a variety of challenges after diagnosis. Around five to eight% of the global population suffers from at least one of over a hundred autoimmune diseases, making it the third most common group of conditions [1]. Of these, 2.8 million (0.034%) worldwide are people with multiple sclerosis (pwMS) [2, 3]. PwMS undergoing immunosuppressive therapy are at increased risk of contracting infections due to this therapy [4]. In addition infections can exacerbate MS symptoms and increase the risk of mortality, thus adequate vaccination protection and complete immunization are required [4–6]. In Germany, recommendations for vaccinations are published by the Standing Committee on Vaccination (Ständige Impfkommission, STIKO) at the Robert Koch-Institute with additional recommendations for chronic diseases [7]. Ideally, all necessary vaccinations should be completed or refreshed two to four weeks before starting immunotherapy, however, this strategy is not possible in all patients due to increased disease activity at first diagnosis. In these cases, treating physicians need to check and complete vaccination status after treatment initiation [8].

There are numerous essential vaccinations for patients undergoing immunotherapy [9–12]. As patients may be concerned about lack of safety and effectiveness, according to the literature available to date, they decide against vaccination, and lower vaccination rates have been reported in pwMS in this context [8, 12, 13]. This is why information from physicians is essential, as patients are not always familiar with all the necessary vaccinations and are more willing to be vaccinated as soon as they are sufficiently informed [14].

In this paper we illustrate the reasons given by pwMS, neurologists and general practitioners (GPs) for vaccination uptake that is described as lower than recommended in previous studies, by answering the research question “What barriers and facilitating factors do pwMS and physicians treating them face, when deciding to vaccinate or not?“. Furthermore, we present possible approaches to improve vaccination acceptance and to support the completion of recommended vaccination schedules in pwMS.

## Methods

The presented study is a sub-project of the project “VAC-MAC: **VAC**cination of MS/Arthritis/Colitis patients” which, in addition to **M**S, is also investigating inflammatory rheumatic diseases (e.g. rheumatoid **a**rthritis) and chronic inflammatory bowel diseases (e.g. **C**rohn’s disease). The project is funded by the Innovation Fund of the Federal Joint Committee (Innovationsausschuss des Gemeinsamen Bundesausschusses (G-BA) in Germany and the study was retrospectively registered at the German Register of Clinical Studies (DRKS-ID: DRKS00031559). A positive vote of the Ethics Committee of the Medical Faculty of the Ruhr University Bochum has been obtained (see below).

The aim was to interview 30 people in total: five neurologists, ten GPs, and 15 pwMS. We expected to reach thematic saturation more quickly with neurologists than with GPs, since GPs’ experiences in vaccination might cover a more diverse range, while neurologists might be able to provide more specific statements based on more specialized experience. To select patients, we drew on our experience from previous research, in which thematic saturation was achieved with 15–20 patients, particularly for such specific topics. Our inclusion criteria for physicians were based on their medical specialty. Patients were required to be at least 18 years old, have one of the conditions we were studying, be fluent in German, and have the mental capacity to participate in an interview. For the recruitment, physicians from the project management of the VAC-MAC consortium received brochures and distributed them to potential participants, including patients and colleagues eligible for the study using a snowball recruitment strategy. In addition, the interviewed physicians were asked to forward the information to their patients. Overall, a purposeful sampling approach was chosen with the goal of recruiting stakeholders (physicians and patients) who, according to their own reports, had experience with MS or vaccinations [15].

Participants were able to contact us by phone or e-mail and received comprehensive initial information about the study, including a consent form, data protection guidelines, and a questionnaire to collect demographic data. On request, a telephone call was offered in advance to clarify further questions and schedule an interview appointment. All participants gave their written informed consent and received an expense allowance.

Between March and December 2023 interviews were conducted by AS, CF and RL (sociologist, M.A., health services researcher, M.Sc. and health scientist, Dr. biol. hum., respectively) using a semi-structured interview guideline developed by the qualitative research team, informed by a literature review, and reviewed collaboratively within the research team [16, 17]. A formal pilot test was not documented.

The researchers took care to select a meaningful opening question that provided participants with a motivating incentive to share their stories. The semi-structured interviews with all participants covered topics such as vaccination recommendations and their implementation, the decision-making process, and their expectations for the future. The planned sample size of 30 participants was achieved. A thematic saturation concluded the recruitment of further participants [16, 18]. Thematic saturation was considered to have been reached when no new statements were made in the interviews. Despite the relatively small number of participants, this was achieved within five to fifteen interviews. As we had expected, the number of different groups initially planned proved to be successful.

Depending on the participants’ preference, interviews were conducted by telephone, via video call, or face-to-face at the participants’ homes or in the universities’ facilities and lasted about 13 and 40 minutes. Audio recording devices were used during face-to-face and telephone interviews. During the online interviews the software Zoom Video Communications, Inc. and their embedded recording tool was used. The recorded interviews were transcribed and pseudonymized by a transcription agency. The interviews were analyzed by AS using Kuckartz’s Qualitative Content Analysis [19]. A deductive coding system in the MAXQDA 2022 software was initially used by coding the first three interviews [20]. Coding was conducted by one researcher (AS). No independent double coding or assessment of intercoder agreement was performed. During the further coding process, additional inductive codes were added to adequately reflect the richness of the data collected [20]. In addition, coding decisions and emerging themes were discussed in biweekly multidisciplinary team meetings (peer debriefing). These discussions provided opportunities to critically reflect on coding decisions and interpretations from different disciplinary perspectives. The researcher was not blinded to participant group during the analysis, as the group affiliation was apparent from the interview context. To enhance the trustworthiness of the findings, a form of member check was conducted: selected interview quotes and interim results were presented in webinars to interested patients, who were invited to ask questions and provide feedback on the preliminary results [21]. The translated coding tree and interview guideline can be found in the appendix.

A demographic questionnaire was used for patients and included age, gender, date of disease diagnosis, and vaccinations received during the previous ten years according to STIKO recommendations [7]. The questionnaire should be completed before the interview. Clinicians were asked about age, gender, medical specialty, and professional experience. Semi-structured interviews for all participants included the recommendation and implementation of vaccinations, the decision-making process, and their wishes for the future.

The reporting of our study was guided by the COREQ Checklist. The completed COREQ checklist has been added as an appendix to provide greater transparency regarding methodological reporting.

The interview quotes displayed below were translated from German into English by the project team.

## Results

A total of 30 interviews were conducted to obtain the attitudes of patients, GPs and neurologists about vaccination in pwMS. No participants refused to be interviewed when contacted. Since the selection of participants was based on a prior survey of interest conducted by the physicians involved in the project, contact was made only with potential participants who had already indicated that they were available for an interview. Table 1 shows the results of the demographic data of the participants.

**Table 1:** Demographic variables of the interview participants

**Characteristics**		
Gender, neurologists ***n (%)***		
Female		5 (100)
Male		0 (0)
Gender, GPs ***n (%)***		
Female		4 (40)
Male		6 (60)
Gender, patients ***n (%)***		
Female		10 (67)
Male		5 (33)
Workplaces of Neurologists ***n (%)***		
Practice		1 (20)
Hospital		2 (40)
Medical Care Center		2 (40)
Age, neurologists ***mean (min-max)***		43 (32-52)
Age, GPs ***mean (min-max)***		53 (40-67)
Age of patients ***mean (min-max)***		43 (21-59)
Age of patients at disease diagnosis ***mean (min-max)***		30 (15-46)
Duration of MS, years, ***mean (min-max)***		13 (1-28)

**Fig. 1:**
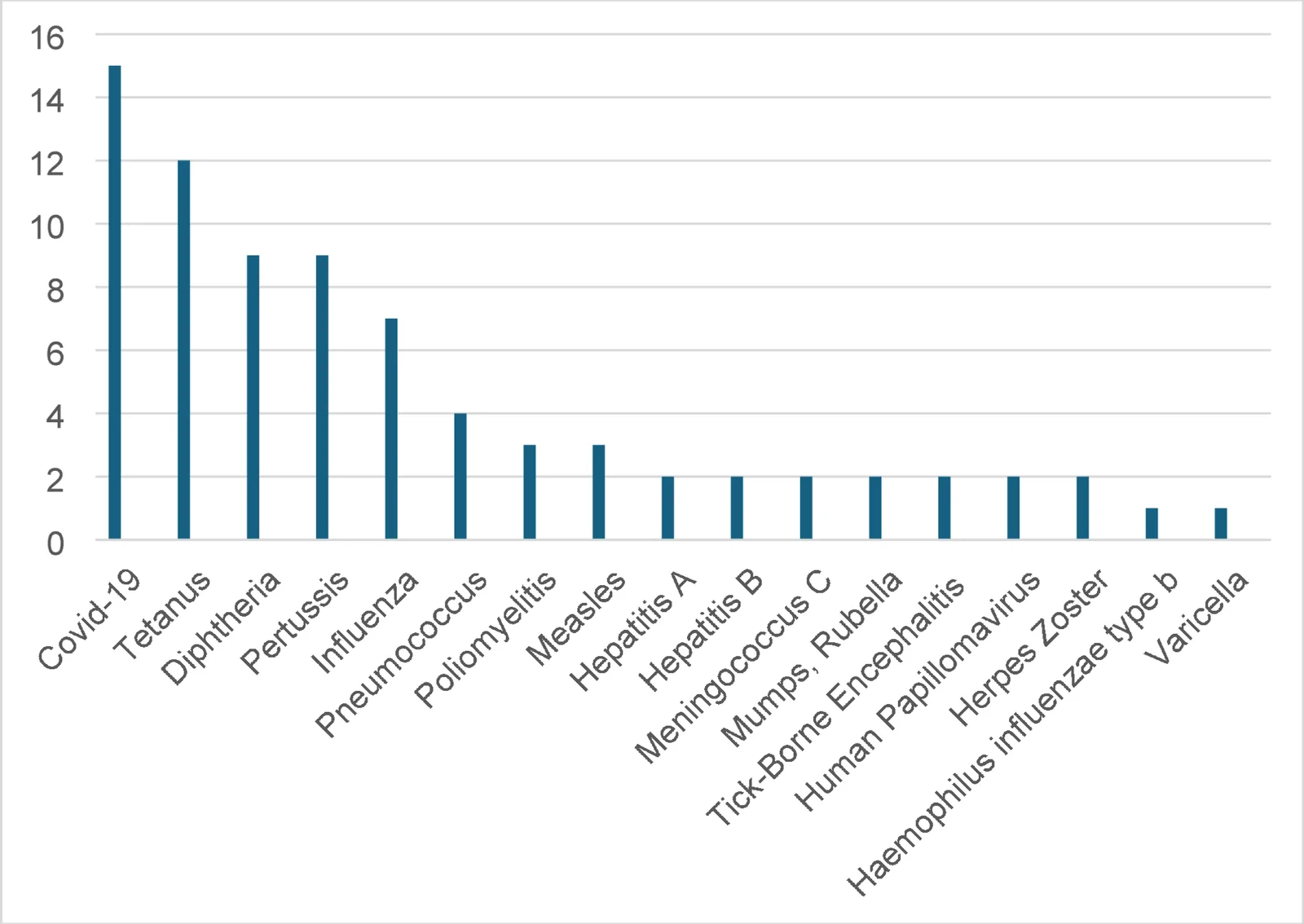
Frequency of vaccinations in *n* = 15 pwMS in the past 10 years

Most of our patients had received vaccinations during the past ten years, particularly those included in the recommended basic immunization schedule (see Fig. 1). All participants had received the COVID-19 vaccination, and the most frequently administered vaccine was the Tetanus vaccine. In contrast, the vaccinations recommended by STIKO for pwMS—influenza, herpes zoster, and pneumococcal—were not as common. 

The analysis of the semi-structured interviews regarding factors that participants associated with their vaccination decisions focused on the following main topics: (a) Information, concerns, and vaccination attitudes among patients, (b) Responsibilities for vaccination advice, implementation, and patients’ experiences, and (c) Demand for exchange between physicians.

### Information, concerns, and vaccination attitudes among patients

The first topic concerns the challenges of coordinating and providing information to patients after diagnosis. It highlights the varying experiences of patients, neurologists and GPs. Patients reported different levels of information and different concerns regarding vaccination. Some patients were well-informed, had already received information from their physician, and in some cases had even received informational materials. Others reported concerns about vaccinations, particularly regarding potential side effects, the possibility of disease relapse, and misinformation encountered online. Patients expressed a desire for information materials provided immediately after diagnosis. These information materials should contain clear information about which vaccinations are appropriate for patients and when. One patient emphasized that it is unrealistic to expect all relevant physicians to have all the relevant information:

*Patient 03: “Yes*,* everything was fine as it was*,* but a guide like this at the beginning*,* what would be useful for you? Perhaps something that would be given to patients directly after diagnosis. […] That this is simply formulated more clearly.”*

Contrary to this opinion, a few patients also report that they were given information materials by their neurologists that contained exact times for vaccinations before and after treatment and report that their vaccination status was checked before new medication was administered. It is pointed out that any vaccination gaps should be closed by the GPs before the start of treatment:

*Patient 06: “And now at the last vaccination I was given a document for my GP*,* who does all my vaccinations*,* […] how many weeks before I can be vaccinated and how many months afterwards*,* I can only be vaccinated again.”*

In addition, a GP reported that patients often seek information online, which can lead to an accumulation of misinformation and further concerns. Neurologists described a wide range, from patients who are highly engaged and well informed to patients whose views are shaped by incorrect information from online sources:

*Patient 08: “But with every vaccination*,* there is certainly also a part of it: ‘Well*,* let’s see what it does to me now’. And that’s simply the worry about side effects or a relapse*,* which I would now also put under side effects*,* you know*,* from renewed disease activity.”*

*Neurologist 03: “In general*,* I would say that patients with chronic illnesses have been dealing with it for a long time. […] Or with*,* on the flipside of the coin*,* a total therapy and vaccination phobia based on false facts*,* false facts that they pull from the internet on some forum*,* I don’t know where.”*

*GP 09: “The MS patients*,* […] are certainly the most difficult when it comes to vaccination. Because there are many myths circulating in the MS community about vaccination*,* […] for example that MS is triggered by vaccinations or that vaccinations cause MS relapses.”*

Due to missing or false information, one patient was worried about possible side effects, such as relapses. He/she pointed out that these concerns can lead him/her to refuse certain vaccinations or not consider them necessary. A neurologist shared her experience with pwMS and emphasized that vaccinations generally do not cause worsening of MS symptoms and can even help prevent infections that could worsen the disease.

### Responsibilities for vaccination advice, implementation, and patients’ experiences

Another issue shows the importance of vaccination education and an active approach by the physicians. The GP is often considered the primary point of contact for vaccinations. On the one hand, one patient states that their neurologist never checked her vaccination status. One patient reported that in her own experience none of her treating physicians had raised the topic of vaccination status with her:

*Patient 08: “Neither my GP*,* nor my gynecologist*,* nor my neurologist*,* which I consider the worst thing*,* actively pointed out to me once: ‘Mrs. X*,* what is your vaccination status?’. It was my own contribution […].”*

In contrast, neurologists and GPs report that it is necessary to check the vaccination status of patients before prescribing immunosuppressive drugs and to discuss this if necessary:

*Neurologist 01: “I always say*,* […] that it is recommended to follow the current STIKO recommendation regarding vaccinations. And that they [the patients] go through the vaccination record with the GP and see what is still pending.”*

*Neurologist 03: “In any case*,* the first thing that comes to mind is the adjustment of MS patients to new medication. It is particularly important to clarify live vaccinations in advance and to go through the vaccination schedule with the patient beforehand.”*

Another topic focuses on the question of which doctors are best suited to vaccinate pwMS. There are different opinions and considerations in this regard. Patients said that they prefer their neurologists for vaccination as they are familiar with current (interactions of) medications and possible side effects. They believe that neurologists are generally better informed and more competent than GPs about MS and its immunosuppressant treatment:

*Patient 13: “Well*,* I think that the GP is in the right place. And I once asked her whether it would be possible to have a vaccination consultation. But I felt very out of place because the connection to immunosuppressants was simply completely missing.”*

Some patients had the impression that their GP was less familiar with MS specific vaccination questions than their neurologist. However, some patients believe that their GP is responsible for vaccination as he/she carries out all vaccinations and has long-term overview of their history best, e.g. their co-morbidities.

### Demand for exchange between physicians

Furthermore, there seems to be a lack of communication between physicians. Once again, this section encourages the maintenance of records regarding vaccinations and the exchange of information among physicians. One patient reported never having experienced physicians exchanging information and described often being the person who carried information between practices. This can lead to fragmentation of information, especially when it comes to routine treatments:

*Patient 14: “I’ve never experienced doctors exchanging information with each other*,* though maybe I haven’t noticed. But I’m always the person*,* as the patient who then carries the information from A to B. […] But there are somehow fragments of information everywhere.”*

One neurologist suggests using a prepared sheet of paper, like the information materials requested by patients:

*Neurologist 02: “But I think it would be very helpful to have a prepared sheet of paper […] and which the GP should then perhaps check. Because you could then give it to the patients. They could take it to their GP. The GP would know directly what the issue is.”*

In addition, one neurologist reported a back-and-forth between GPs and neurologists regarding vaccination issues. Overall, it is emphasized that both GPs and neurologists should play a role in vaccinating pwMS:

*Neurologist 02: “And I think […] that*,* vaccination issues in particular are always such a ping-pong between doctors. So*,* patients are sent back and forth from the GP to the neurologist*,* from the neurologist to the GP*,* to the neurology outpatient clinic and back again. In my opinion*,* this is mainly since the GP is understandably not familiar with Immunotherapeutics and the neurologist either does not vaccinate or is not familiar with the vaccination calendar.”*

A neurologist points out that doctors’ time, especially time for medical advice and education, is valuable:

*Neurologist 03: “Yes*,* as I just mentioned*,* medical time*,* especially medical speaking time*,* is a precious commodity and time to talk about medical issues should be rewarded more than the additional technical examination. In other words*,* more speaking time in relation to information*,* including vaccination information. After so many years in the profession*,* it would feel like a luxury to really have more time for the patient in individual cases.”*

Since the COVID-19 pandemic was still recent, the interview guideline also included questions about changes in the attitudes of patients and physicians toward vaccinations in general. However, in most cases, patients stated that neither their own attitudes nor those of their physicians had changed. Physicians also reported that there were only a few people who opposed vaccinations.

## Discussion

Previous studies have predominantly examined vaccination uptake, vaccination willingness, and factors associated with vaccine hesitancy among pwMS [11, 13, 22]. While these studies provide important insights into patients’ attitudes towards vaccination, less is known about how vaccination-related information and decision-making are experienced and negotiated within routine care. In particular, the perspectives of patients, neurologists, and GPs have rarely been examined together within the same qualitative study.

Bringing these perspectives together allows us to explore how responsibilities for vaccination counselling and implementation are distributed across healthcare professionals and how communication between care providers may shape the information patients receive about vaccination and their subsequent decisions.

Vaccination services are organized differently across countries, and in some settings vaccinations are also offered in pharmacies [23]. In Germany, pharmacies may obtain special permits allowing them to administer vaccinations, but most vaccinations are still administered in GPs’ practices [24]. Our study therefore focused on the outpatient practice setting.

The interviews shed light on the opinion and concerns of pwMS and their GPs and neurologists regarding vaccinations. The statements quoted here emphasize the importance of improved medical communication and education, especially in connection with vaccinations for pwMS and other chronic diseases.

During therapy, pwMS have an increased risk of contracting various infectious diseases, which in turn can increase morbidity or mortality [4, 8]. Therefore, it is essential to ensure that the vaccination status is checked as far as possible and to carry out any necessary vaccinations [4, 10, 25]. Nevertheless, some of the interviewed pwMS are reluctant to be vaccinated. Most of the patients we interviewed had received several recommended vaccinations during the previous ten years, although some expressed concerns regarding potential side effect or disease relapse Some vaccinations, such as measles, mumps, rubella, and human papillomavirus, may be underrepresented in our results because vaccinations received prior to the 10-year observation period were not assessed. These vaccinations are commonly administered during childhood or adolescence and may therefore have been received before the period covered by our questionnaire. Consequently, we cannot determine from our data whether participants had previously received vaccinations or had existing immunity against specific infections.

In summary, the present study showed that patients have a comprehensive need for information. Due to their medical condition, they may be unsure about which vaccinations are necessary and what side effects there might be. The interviewed pwMS are well informed in many cases but can also be exposed to misinformation. Participants described communication between physicians of different specialties as an aspect that, from their perspective, influenced how vaccination questions were addressed. Some interviewed patients expressed a preference for receiving vaccination counselling from their neurologist, but they tend to see the responsibility as lying with their GP. Both neurologists and GPs described that the time available in routine consultations for comprehensive vaccination counselling is limited. Neurologists therefore would like to present patients with an information sheet with the most important vaccinations and their necessary time frames, which can be presented to the physicians involved.

The literature to date has shown that certain vaccines can raise doubts and uncertainties in pwMS. Some patients fear a relapse if they are supposed to be vaccinated [13, 22, 26]. Although statements about such fears were made in our interviews, we were uncertain where this information came from because the participants did not give any specific explanations or could not name any precise sources other than the “Internet”. One possible explanation discussed by participants was that patients may encounter misleading vaccination-related information online [27–32]. However, our study did not investigate patients’ information-seeking behavior or the sources of the information they encountered. In addition, patients undergoing alternative medical treatment have the highest rate of vaccination refusal [9]. All patients we interviewed had been vaccinated against COVID-19 and did not express any explicit concerns about this vaccination. In line with this, current evidence indicates that vaccinations are unlikely to trigger a worsening of MS symptoms [4, 8, 12, 13, 33, 34]. In fact, infections have been associated with an increased risk of relapses and increased lesion activity on MRI [10]. Currently, immunization through vaccination is therefore one of the most effective methods of preventing potentially serious infections [12].

Possible concerns raised by patients should therefore be treated with trust and care by their physicians, whether neurologists or GPs [4]. This was also the preference of the patients we interviewed. The same applies to the provision of information material containing the most relevant information and data on the vaccinations that need to be administered. On the one hand, the vaccination status should be checked, but on the other hand, comprehensible information material should also be provided for patients [4]. Our interviews also showed that communication between the different physicians treating a patient is of crucial importance. In the best-case scenario, neurologists check the vaccination status, but in most cases, it is up to the GPs to administer the vaccination [13, 25]. Patients in our study often prefer neurologists for vaccination counseling due to their MS-specific knowledge, but GPs are more likely to have the possibility to administer vaccinations and patients prefer the GPs’ knowledge of their entire medical history. The information material mentioned can be beneficial in this case, which is presented to the GP to indicate which vaccinations are relevant for pwMS and the vaccination status can be shown to the neurologist. To adapt this to the wishes expressed in the interviews, we have created a factsheet and a medical letter that can be downloaded from our website www.vac-mac.de (German language). The information provided there can help the neurologist to present and select the recommended vaccinations briefly. The patient could then present the factsheet to the GP after the neurologist has marked the necessary vaccinations and the GP marks the vaccinations given. Patients could thus ensure that their physicians are up to date and inform themselves on the homepage and use the factsheet. The information and resources presented on the website are based on the study results and therefore represent a preliminary form of support that patients have frequently requested and that we are confident can be helpful. Whether the materials are actually useful for all those involved would require further evaluation.

### Strengths and limitations

This study’s strength lies in its qualitative design, which integrates the perspectives of pwMS, GPs and neurologists. In addition, we were able to gather a large number of insightful interview quotes that illustrate various aspects of pwMS’ willingness to get vaccinated. This approach enables a comprehensive understanding of the multi-layered barriers and facilitators associated with vaccination. By applying qualitative content analysis according to established methodological standards, a systematic and transparent analysis process was ensured. This allowed us to address a clinically relevant topic and draw implications for clinical practice that emphasizes structured communication among the various physicians, thereby closing an important gap in the implementation of vaccinations for pwMS.

However, some limitations should be noted. Although the strategy of purposive sampling is suitable for qualitative research, it may limit the transferability of the results. Although the sample size is small, it nevertheless allowed us to achieve thematic saturation and thereby gain insight into the subjective perceptions of patients, GPs, and neurologists. The patients we interviewed reported that they were adequately vaccinated. Therefore, it cannot be ruled out that patients who were willing to get vaccinated were also more likely to participate in the study, which may have led to selection bias. Nevertheless, our interviews provided numerous insights into barriers to and facilitators of vaccination. Furthermore, clinical characteristics such as disability status and current or previous disease-modifying therapies were not assessed in our study. As these factors may influence vaccination recommendations and attitudes towards vaccination, their absence should be considered when interpreting our findings. A triangulation with quantitative insights or observations might have provided even deeper insights but was not the objective of this study. Furthermore, our conclusions are limited exclusively to the German healthcare system and therefore cannot be applied to other healthcare systems. Finally, the qualitative design does not allow for statistical generalization of the results.

## Conclusion

Our study highlights key barriers and facilitating factors regarding vaccination in pwMS. While many patients are well-informed and motivated, uncertainties, misinformation, and unclear responsibilities were described by participants as factors that can make vaccination decisions more difficult. Improved communication between GPs and neurologists, as well as structured information materials for patients, could enhance vaccination acceptance and improve vaccination coverage in pwMS. The information sheet developed in this project is provided in the appendix as an English translation.

## Supplementary Information

Below is the link to the electronic supplementary material.

Supplementary Material 1: Translated Coding tree_patients

Supplementary Material 2: Translated Coding tree_physicians

Supplementary Material 3: Translated guideline_patients

Supplementary Material 4: Translated guideline_physicians

Supplementary Material 5: VAC-MAC Consortium

Supplementary Material 6: VACMAC_Factsheet_EN

## Data Availability

The data sets created and/or analyzed in the current study are not publicly available for reasons of anonymity of the participants but can be requested from the corresponding author upon reasonable request.

## Abbreviations

MS: Multiple Sclerosis
pwMS: People with multiple sclerosis
STIKO: Ständige Impfkommission (Standing Committee on Vaccination)
VAC-MAC: –VACcination of MS/Arthritis/Colitis patients
GBA: Gemeinsamer Bundesausschuss (Federal Joint Committee)
GP: General Practitioner

## Abbreviations

MS: Multiple Sclerosis
pwMS: People with multiple sclerosis
STIKO: Ständige Impfkommission (Standing Committee on Vaccination)
VAC-MAC: –VACcination of MS/Arthritis/Colitis patients
GBA: Gemeinsamer Bundesausschuss (Federal Joint Committee)
GP: General Practitioner
