# Supplementary material for: Attitudes towards vaccination in multiple sclerosis: insights from patients, neurologists, and general practitioners within a qualitative study
Source: BMC Neurol. 2026 Nov 3;26:697. doi: 10.1186/s12883-026-05590-1 (PMC13733131; doi:10.1186/s12883-026-05590-1)
Supplement: Supplementary file 2 [file 12883_2026_5590_MOESM2_ESM.docx]

List of Codes

| **Lift of Codes** | **Frequency** |
| --- | --- |
| Codesystem | 1009 |
| Practice features | 19 |
| Interaction with patients (treatments - general) | 10 |
| Total number of patients | 2 |
| Number of patients without MAC | 4 |
| Number of patients with MS/CIRD/CED | 15 |
| Cooperation / exchange with other specialists | 51 |
| Delegating to other physicians | 21 |
| Treatments of MS/CIRD/CED | 21 |
| Examinations with MS/CIRD/CED | 5 |
| Open vaccination consultation hours | 2 |
| Special consultation hours for MAC | 10 |
| MAs carry out vaccinations | 13 |
| Physician carries out vaccinations themselves | 22 |
| Attitude towards vaccinations | 7 |
| Knowledge and abilities | 0 |
| For vaccination recommendation | 32 |
| During vaccination | 6 |
| Experience with vaccine damage | 6 |
| Advantages of vaccinations | 26 |
| Responsibility for vaccinations | 40 |
| Question about vaccination certificate / vaccinations | 34 |
| Researching information about vaccinations | 10 |
| Vaccinations depending on treatment | 48 |
| Observe medical history | 5 |
| Education about vaccinations | 16 |
| Respecting the autonomy of patients | 8 |
| Type of vaccinations | 0 |
| Hepatitis | 6 |
| Travel immunizations | 10 |
| Measles | 1 |
| Meningococcus | 1 |
| Chickenpox (varicella) | 4 |
| Covid-19 | 32 |
| Influenza | 22 |
| Yellow fewer | 1 |
| TBE | 13 |
| (Pneumavax) Pneumococcus | 24 |
| Shingrix (shingles) | 33 |
| Repavax (T,D,K,K) | 17 |
| Advise patients about vaccinations | 4 |
| Vaccinations that “slip through” | 1 |
| Difference between MAC/non-MAC vaccination recommendation | 15 |
| Advice on vaccination | 21 |
| “Persuade” patients | 15 |
| Address during consultation hours | 30 |
| Handing out information material | 12 |
| Software (transfer of vaccination status) | 7 |
| Reminder of vaccination | 5 |
| Patients' own initiative | 18 |
| Open mindedness towards vaccinations | 27 |
| Anti-vaccination | 9 |
| Worries / concerns of the patients | 32 |
| How physicians deal with concerns | 22 |
| Fear of relapses | 5 |
| Strong rejection of vaccinations / conspiracy theorists | 10 |
| Vaccination recommendation for immunosuppressed patients | 8 |
| Determine vaccination titre | 3 |
| Considering the timing of vaccinations | 23 |
| Evaluation of one's own skills | 18 |
| Rating competence of other physicians | 8 |
| MS | 8 |
| CED | 4 |
| CIRD | 7 |
| Conclusion | 0 |
| Requests for remuneration / time / training | 29 |
| Requests for information material | 6 |
| General wishes for MAC patients | 11 |
| Desire for more cooperation with physicians | 15 |
| Wishes regarding vaccinations | 29 |
| Desire for more digitalization | 10 |
