# Supplementary material for: Attitudes towards vaccination in multiple sclerosis: insights from patients, neurologists, and general practitioners within a qualitative study
Source: BMC Neurol. 2026 Nov 3;26:697. doi: 10.1186/s12883-026-05590-1 (PMC13733131; doi:10.1186/s12883-026-05590-1)
Supplement: Supplementary file 3 [file 12883_2026_5590_MOESM3_ESM.docx]

**VAC-MAC: Vaccination and infection rates in multiple sclerosis, chronic inflammatory rheumatic diseases or chronic inflammatory bowel diseases**

**Guideline for MAC patients**

Thank you for your time.

I would like to briefly introduce the topic to you in more detail.

The aim of the interviews is to identify barriers and supporting factors for doctors and patients with autoimmune diseases with regard to vaccinations. In regards to autoimmune diseases, we are looking at multiple sclerosis, chronic inflammatory rheumatic diseases and chronic inflammatory bowel diseases. We also want to get to know individual vaccination strategies of the treating doctors and look at reasons for low vaccination rates in patients with these diseases.

It is about all vaccinations, such as tetanus, measles or influenza, and not just COVID-19.

We are interested in your experiences, so there are no right or wrong answers. Only what you think about the topic is important.

It is important to us that you feel comfortable. This means that if a question makes you uncomfortable or you don't want to answer it, you don't have to.

Do you consent to the interview being recorded using an audio device?

Do you have any questions?

I now turn on the recording device.

Thank you for agreeing to do the interview with me as part of the project and for recording the conversation. We have spoken at detail about the project. We want to find out what experiences you have had with your autoimmune disease in connection with vaccinations.

I'll start with the first question now.

**Topic I: Introduction to the topic**

**General introduction**

- I read in the questionnaire that you were diagnosed with [disease] in [year]. Please tell me about the care you received for your illness, especially with regard to vaccinations.

**Topic II: Information, recommendations and implementation of vaccinations**

- Which specialist is responsible for you and your illness?
  - How often do you see your specialist on average?
  - In what context does your specialist care for you? (e.g. special consultation hours, special training courses)?
- Who advises you on vaccinations? *(Ask for specialist group)*
- How does the consultation take place?
  - How did the doctors communicate with you?
  - How detailed is the consultation?
  - How does the doctor handle this?
  - What information or materials have you received?
- What advantages do you see in vaccinations?
- What worries and concerns do you have about vaccinations?
  - Do you ask your doctor about this?
  - How do doctors deal with this?
- The vaccinations you indicated in the questionnaire: who carried them out? *(Ask for group of specialists)*
- If you look at the advice and implementation of the vaccination now: how happy are you about it?
- Which group of specialists would you prefer to be advised by? For what reasons?
- Which group of specialists would you prefer to be vaccinated by? For what reasons?

**Topic III: Decision-making**

- How do you decide whether or not to get vaccinated?
  - *Address vaccinations in the questionnaire*
- How do you decide whether or not to have a vaccination - regardless of your illness? *(general or specific opposition to vaccination)*
  - On which factors do you base your decision?
- We have already talked about the benefits and concerns of vaccinations. Where do you inform yourself about the advantages and disadvantages of vaccination?
  - Why do you seek information from these sources?
- When deciding whether or not to get vaccinated, what role does your own autoimmune disease play in this context?
  - *e.g. concern about worsening due to vaccination*
- What role does your treatment, e.g. medication, play in your decision to be vaccinated?
- To what extent did you involve your social environment in the decision to be vaccinated or not?
- How did people in your community react to the fact that you (did not) get vaccinated?
  - Why do you think those people around you behaved in this way?
  - What did that do to you?
- How has the pandemic changed your attitude?
- How has the attitude of your doctors changed as a result of the pandemic?

**Topic IV: Wishes**

If you now think about everything we have discussed:

- What do you wish for your future care and that of other patients with [disease]?
  - What wishes do you have regarding vaccinations?
  - Who would you like to receive this from?

**Topic V: Conclusion**

- I have now asked all the questions that are important to us. Is there anything else we haven't discussed so far that you would like to add?

Switch off the recording device.

- What was it like for you?
- Do you have any questions?
