# Supplementary material for: Attitudes towards vaccination in multiple sclerosis: insights from patients, neurologists, and general practitioners within a qualitative study
Source: BMC Neurol. 2026 Nov 3;26:697. doi: 10.1186/s12883-026-05590-1 (PMC13733131; doi:10.1186/s12883-026-05590-1)
Supplement: Supplementary file 4 [file 12883_2026_5590_MOESM4_ESM.docx]

**VAC-MAC: Vaccination and infection rates in multiple sclerosis, chronic inflammatory rheumatic diseases or chronic inflammatory bowel diseases (MAC diseases)**

**Guideline for doctors**

Thank you very much for taking the time.

I would like to briefly introduce the topic to you once again.

The aim of the interviews is to identify barriers and supporting factors for treating physicians and patients with autoimmune diseases towards vaccinations.

We also want to get to know individual vaccination strategies of the treating physicians and examine the reasons for low vaccination rates in these patients.

The focus will be on all vaccinations, such as tetanus, measles or influenza, and not just COVID-19.

We are interested in your experiences, so there are no right or wrong answers. Everything you might think about the topic is important.

It is also important to us that you feel comfortable. This means that if a question makes you uncomfortable or you don't want to answer it, you don't have to.

Do you consent to the interview being recorded using an audio device?

Do you still have any questions?

I now switch on the recording device.

Thank you for agreeing to conduct the interview with me as part of the project and to record the conversation. We have spoken about the project in detail.

We want to examine what experiences you have had with vaccinations in connection with autoimmune diseases.

I'll start with the first question.

**Topic I: Introduction to the topic**

**General introduction**

- How do you come into contact with vaccinations in your day-to-day work?
  - Are you carrying out vaccinations yourself?
- How do you generally decide whether or not to recommend vaccination, irrespective of MAC patients? *(general or specific opposition to vaccination)*
- Please tell me about your day-to-day work with the patients in your practice, specifically with regard to vaccinations.
  - I have seen in the questionnaire that you care for [number] patients with [disease]. What special features characterize the care of these patients with regard to vaccinations?
  - In what setting do you care for the patients (e.g. special consultation hours, special training)? What is your opinion on this, is it suitable to this extent?

**Topic II: Recommendation and implementation of vaccinations**

Please recall the consultation hours with the MAC patients for whom vaccination is an option.

- *When treating several MAC groups:* For which of the three conditions do you recommend vaccination?
  - What advantages do you see in vaccinations for people with autoimmune diseases?
  - What challenges do you see with vaccinations for people with autoimmune diseases?
- At what stage of the disease do you recommend a vaccination?
  - Do you actively approach patients?
  - Which vaccination strategy do you use, e.g. in the context of immunosuppressive medication?
    - What does this vaccination strategy look like? What do you pay attention to? How do you proceed?
- What differences do you make in the vaccination recommendation between MAC and non-MAC patients?
- What knowledge and skills do you use to recommend vaccinations?
- What knowledge and skills do you use to carry out vaccinations?
- Who do you think is responsible for carrying out vaccinations?
  - What do you think are the reasons for this?
- Which kind of exchange do you have with GPs?

Describe what you would like to have and from whom? (e.g. knowledge, training opportunities, remuneration, time/personnel capacities)

**Topic III: MAC patients**

- How receptive are MAC patients to the topic of vaccination? (communication level)
  - Request: How do you determine the existing/not existing open-mindedness?
- With what worries/concerns do patients turn to you?
  - Request: How do you deal with that?

**Topic IV: Wishes and conclusion**

If you now think about everything we have discussed:

- What would you like to change in the future care of patients with autoimmune diseases?
  - Who would you like to receive this from?
- I have now asked all the questions that are important to us. Is there anything else we haven't discussed so far that you would like to add?

Switch off the recording device.

- What was it like for you?
- Do you have any questions?
