# Supplementary material for: Attitudes towards vaccination in multiple sclerosis: insights from patients, neurologists, and general practitioners within a qualitative study
Source: BMC Neurol. 2026 Nov 3;26:697. doi: 10.1186/s12883-026-05590-1 (PMC13733131; doi:10.1186/s12883-026-05590-1)
Supplement: Supplementary file 5 [file 12883_2026_5590_MOESM5_ESM.docx]

**VAC-MAC CONSORTIUM**

Kerstin Hellwig (5)*, Theresa Oganowski (5), Thomas Grüter (5,9), Nina Timmesfeld (2), Marianne Tokic (2), Jale Basten (2), Robin Denz (2), Katharina Meiszl (2), Romy Lauer (2), Ingo Meyer (12), Katja Blaschke (12), Heike van de Sand (12), Carolin Heinen (12), Stephanie Stock (10), Dusan Simic (10), Arim Shukri (10), Uta Kiltz (3, 11), Maria Zacharopoulou (3, 11), Horst Christian Vollmar (1), Anastasia Suslow (1), Ina Carola Otte (8), Chantal Flemm (4), Andreas Stallmach (6), Anika Franz (6), Ursula Marschall (7), Joachim Saam (7), Catharina Tiemann (7), Kathrin Grochau (7), Katja Matthias (13), Daniel Salas (13), Jill-Marie Fix (13), Sarah Peitz (13), Philipp Lutz (13), Iris Hermanski (13)

1 Ruhr University Bochum, Medical Faculty, Institute of General Practice and Family Medicine (AM RUB), Bochum, Germany

2 Ruhr University Bochum, Department of Medical Informatics, Biometry and Epidemiology, Bochum, Germany

3 Ruhr University Bochum, Bochum, Germany

4 Department of Geriatric Medicine, Marien Hospital Herne, Ruhr University Bochum, Herne, Germany

5 Ruhr University Bochum, Department of Neurology, St. Josef Hospital, Bochum, Germany

6 Jena University Hospital, Department of Internal Medicine IV (Gastroenterology, Hepatology and Infectious Diseases), Jena, Germany

7 BARMER Institute for Health System Research, Department Medicine and Health Services Research, Wuppertal, Germany

8 Ruhr University Bochum, Department of Health Services Research, Institute of Diversity Medicine, Bochum, Germany

9 Department of Neurology and Stroke Unit, Klinikum Lippstadt, Lippstadt, Germany

10 University of Cologne, Institute of Health Economics and Clinical Epidemiology (IGKE), Faculty of Medicine and University Hospital Cologne, Cologne, Germany

11 Rheumazentrum Ruhrgebiet, Herne, Germany

12 University of Cologne, PMV Research Group, Medical Faculty and University Hospital Cologne, Cologne, Germany

13 TAKEPART Media + Science GmbH

*Nominated consortia representative:

Prof. Dr. Kerstin Hellwig, Ruhr University Bochum, Department of Neurology, St. Josef Hospital, Grundrunstraße 56, 44791 Bochum, Germany. Tel.: +49 234 509 0, E-Mail: kerstin.hellwig@rub.de
