# Supplementary material for: Attitudes towards vaccination in multiple sclerosis: insights from patients, neurologists, and general practitioners within a qualitative study
Source: BMC Neurol. 2026 Nov 3;26:697. doi: 10.1186/s12883-026-05590-1 (PMC13733131; doi:10.1186/s12883-026-05590-1)
Supplement: Supplementary file 6 [file 12883_2026_5590_MOESM6_ESM.docx]

# Factsheet on Vaccination in Immune-Mediated Diseases

At vac-mac.de, you will find information, podcasts, and webinars about vaccinations for people with the most common immune-mediated diseases — multiple sclerosis, rheumatoid arthritis, and chronic inflammatory bowel disease.

The website is aimed at patients and treating physicians.

We thank everyone for their support and for making VAC-MAC possible.

The Standing Committee on Vaccination (STIKO) recommends also vaccinating close contacts of patients with immune-mediated diseases.

https://www.vac-mac.de

## Why is vaccination especially important in immune-mediated diseases?

Patients with an immune-mediated disease and on immunotherapy are at increased risk of infections and severe course of infection. Even under immunotherapy, an infection can worsen the underlying disease and trigger relapses.

Patients with an immune-mediated disease should receive extended vaccination protection. This means that certain vaccinations are recommended additionally or at a younger age than for healthy individuals.

## Do vaccinations work in patients with immune-mediated diseases?

In general, patients without therapy show good immune response with sufficient protection from vaccinations. Depending on the type of immunotherapy, more doses may be needed to achieve protection.

Vaccination is also advisable for patients with immune-mediated diseases and on immunosuppression.

## Do vaccinations trigger immune diseases, relapses, or disease progression?

Vaccinations activate the immune system to build long-term immune defense against infection. However, the risk of relapse or progression after vaccination is low.

Vaccinations far more often protect against relapses caused by infections than they trigger them. Nevertheless, patients may associate fears and concerns with vaccinations. During counseling, there should always be room for discussing these concerns and for joint decision making.

According to current evidence, immune-mediated diseases are not triggered by vaccinations.

## What about pregnancy?

Special vaccination recommendations may apply during pregnancy.

Further information available at: www.vac-mac.de/schwanger

## Can all vaccinations be administered?

Considering the current immune status, all inactivated and RNA vaccines can be given. Under immunosuppressive therapy, live vaccines should not be administered (with few exceptions, e.g., low-dose glucocorticoid therapy: < 10 mg prednisolone equivalent/day).

## When is the right time for vaccination?

• Complete and update vaccinations before starting immunotherapy.
• If disease activity is high, immunotherapy should not be postponed for a vaccination — vaccinations can be given during therapy.
• Avoid vaccinations during periods of high disease activity.
• Preferably vaccinate in the middle of an application interval of the immunotherapy.

## What is VAC-MAC?

Many patients with immune-mediated diseases are insufficiently vaccinated and thus inadequately protected from certain infections. VAC-MAC (VACcination in MS/Arthritis/Colitis patients) is an innovation project that investigates how chronic inflammatory, immune-mediated diseases and their therapies affect infection risk and vaccination willingness. Based on the data obtained, target-group-specific information material is developed to improve vaccination coverage in this vulnerable patient group and to enable optimal healthcare.

## Recommended Vaccinations

| Disease | Recommendation |
| --- | --- |
| Tetanus (lockjaw) | Primary immunization in childhood, booster in adolescence, then every 10 years |
| Diphtheria | Primary immunization in childhood, booster in adolescence, then every 10 years |
| Pertussis (whooping cough) | Primary immunization in childhood, booster in adolescence, once in adulthood |
| Poliomyelitis (polio) | Primary immunization in childhood, booster in adolescence, possibly one booster in adulthood |
| Hepatitis B (liver inflammation) | For risk of severe course; regular antibody testing; booster if Anti-HBs < 100 IU/L |
| Influenza (flu) | Annual booster in autumn |
| Pneumococcal disease (pneumonia) | PCV20 vaccination; after PPSV23, revaccinate with PCV20 after 12 months; after PCV13 + PPSV23, revaccinate with PCV20 after 6 years |
| Herpes zoster (shingles) | Vaccination from age 50, earlier after individual risk-benefit assessment |
| SARS-CoV-2 (COVID-19) | Basic immunity (≥ 3 antigen contacts, ≥ 1 vaccination; titer check if needed), annual booster in autumn recommended |
| Meningococcal disease (meningitis) | MenACWY and MenB vaccination required for hypo/asplenia, complement disorders, complement inhibitor therapy, hypogammaglobulinemia; also for close contact with children or travelers from high risk regions |
| Respiratory Syncytial Virus (RSV, pneumonia) | Once before RSV season with protein-based RSV vaccine from age 75; from age 60 in case of severe chronic disease, acquired immunodeficiency, or residence in nursing homes |

## Disclaimer

As current vaccination recommendations may change, the information provided does not claim completeness or up-to-date accuracy. Current recommendations of the Robert Koch Institute / Standing Committee on Vaccination (STIKO): www.rki.de/impfen-faktenblaetter

We recommend that the vaccination status of patients under planned or ongoing immunosuppression be checked and updated according to the latest STIKO recommendations.

A vaccination letter for your patients can be found here: www.vac-mac.de/impfen
Sources and further information: www.vac-mac.de

Status: September 2024
